# A Competitive Bio-Barcode Amplification Immunoassay for Small Molecules Based on Nanoparticles

**DOI:** 10.1038/srep38114

**Published:** 2016-12-07

**Authors:** Pengfei Du, Maojun Jin, Ge Chen, Chan Zhang, Zejun Jiang, Yanxin Zhang, Pan Zou, Yongxin She, Fen Jin, Hua Shao, Shanshan Wang, Lufei Zheng, Jing Wang

**Affiliations:** 1 Key Laboratory for Agro-Products Quality and Food Safety, Institute of Quality Standards & Testing Technology for Agro-Products, Chinese Academy of Agricultural Sciences, Beijing, 100081, China

## Abstract

A novel detection method of small molecules, competitive bio-barcode amplification immunoassay, was developed and described in this report. Through the gold nanoparticles (AuNPs) probe and magnetic nanoparticles (MNPs) probe we prepared, only one monoclonal antibody can be used to detect small molecules. The competitive bio-barcode amplification immunoassay overcomes the obstacle that the bio-barcode assay cannot be used in small molecular detection, as two antibodies are unable to combine to one small molecule due to its small molecular structure. The small molecular compounds, triazophos, were selected as targets for the competitive bio-barcode amplification immunoassay. The linear range of detection was from 0.04 ng mL^−1^ to 10 ng mL^−1^, and the limit of detection (LOD) was 0.02 ng mL^−1^, which was 10–20 folds lower than ELISA (Enzyme Linked Immunosorbent Assay). A practical application of the proposed immunoassay was evaluated by detecting triazophos in real samples. The recovery rate ranged from 72.5% to 110.5%, and the RSD was less than 20%. These results were validated by GC-MS, which indicated that this convenient and sensitive method has great potential for small molecular in real samples.

Immunological assays have the characteristics of specificity, sensitivity and ease of handling, which has been widely used compared with other detection methods. Many efforts have been made to improve the detection sensitivity, researchers usually couple the target-specific antibodies with various signal amplification strategies including fluorescence dyes, chemiluminescent agents, enzymes, or radioactive isotopes^1^,^2^. The antigen-antibody binding and signal amplification steps are very important for the sensitive detection of antigen molecule^3^. When the level of small molecules sometimes is very low, the sensitivity of immunoassay methods usually does not meet these requirements.

The rapidly emerging research field of nanotechnology provides exciting new possibilities for the advanced development of novel analytical methods^4^,^5^. One major merit of using nanotechnology is that one can control and tailor the properties in a predictable manner to meet the needs of specific applications^6^,^7^. Recently, a novel ultrahigh-sensitivity technique known as the bio-barcode amplification assay based on nanotechnology has attracted substantial research interest in analytical fields^8^,^9^,^10^,^11^,^12^,^13^,^14^,^15^,^16^,^17^,^18^. The barcode assay is a sensitive strategy that takes advantage of short oligonucleotides as surrogate targets in biological detection. Mirkin *et al*.^11^ established a bio-barcode assay to quantify prostate-specific antigen (PSA) based on nanoparticles. The sensitivity of this method was higher than accepted conventional assays for detecting the same target. Mirkin *et al*. then developed a fluorophore-based bio-barcode amplification assay for proteins. This method is more sensitive than immuno-PCR for the systems studied thus far, does not rely on enzymatic amplification, and is less complex^18^. Cao *et al*.^17^ reported a simple and efficient approach for detecting avian influenza virus (AIV) by coupling a fluorophore-DNA barcode and a bead-based immunoassay. Jeung Hee An *et al*.^14^ developed a nanotechnology-based bio-barcode amplification analysis for detecting neurotransmitters using surface-enhanced Raman spectroscopy (SERS), which provides polymerase chain reaction (PCR)-like sensitivity. Most sandwich-type bio-barcode amplification assays have been applied to detect macromolecular substances such as viruses, tumor markers, and cytokines^19^, whereas few reports have focused on small molecules. Furthermore, small molecule (MW < 5000) detection is very important in physiological function research, drug discovery, and detection of veterinary drug residues in foods, etc^20^,^21^,^22^. Therefore, it is very valuable if the bio-barcode amplification assays is used in the quantitative detection of small molecules, such as pesticide, veterinary drugs, environmental pollutant, food additives, small molecules biomarkers. The lack of sufficient binding sites for small molecular antigens and haptens limits its application by sandwiched “antibody-antigen-antibody” structures^6^. By contrast, a competitive immunoassay is appropriate for detecting small molecular antigens.

In order to test the application of the newly development competitive bio-barcode amplification immunoassay method in small molecule detection, we constructed the competitive bio-barcode amplification immunoassay for triazophos, a broad-spectrum organophosphorus insecticide applied for pest control in rice paddies. Hazard and potential risk to human and nontarget species of its residue in food and environment is a growing concern due to its stablity and relatively slow degradation in the environment^23^,^24^,^25^. The conventional detecting approaches such as gas chromatography-mass spectrophotometry (GC/MS) and enzyme-linked immunosorbent assay (ELISA) are not acceptable when the concentration of residues is very low. Thus, in this study, we present the first example of small molecule detection with a competitive bio-barcode amplification immunoassay based on nanoparticles and real-time PCR. As we know, PCR is the most efficient method for DNA detection, and the limit of detection is one copy.

First, we prepared components for the competitive bio-barcode amplification immunoassay as shown in Fig. 1A bio-bar-coded gold nanoparticles (AuNPs) probe labeled antibody was designed by the assembly of antibody and alkylthiol-capped DNA (capture DNA) and bio-code DNA on gold nanoparticles. A magnetic nanoparticles (MNPs) probe was developed by combining magnetic nanoparticles and ovalbumin (OVA) coupled with the small molecules hapten.

Then a direct competitive immunoassay system for small molecules was generated. The AuNPs carries large quantities of barcode strands which could be reflected from the changes of cycle threshold (Ct) values and quantified by real-time PCR. Because of the particle enrichment, a small quantity of target can be converted to a large number of barcodes, allowing this assay to exhibit high sensitivity for targets.

## Results and Discussion

### Characterization of the AuNP probes

Gold nanoparticles were prepared by citrate reduction. The TEM and UV/Vis spectroscopy were used to qualitatively characterise the successful formation of biomolecule-coated AuNPs probes. The UV and visible optical spectra of unmodified and modified gold nanoparticles are shown in Fig. 2A. As expected, the maximum absorbance wavelength of unmodified gold nanoparticles was 518 nm, whereas the maximum absorbance wavelength for modified gold nanoparticles was right-shifted to 526 nm, indicating that the spectral characteristics of the gold nanoparticles was altered due to the particle size changed^26^. TEM imaging (Fig. 2B,C) showed that the diameter of the gold nanoparticles was 15 nm. The unmodified and modified gold nanoparticles did not exhibit agglomeration. A shadow coating was observed after bare AuNPs (Fig. 2B) being coated with antibody and DNA (Fig. 2C) due to the presence of a coating material with a lower electron density. The electrostatic and steric repulsion forces were two major repulsion forces responsible for AuNPs stabilization^27^. The surface charge was measured to determine whether particles with the antibody and DNA would form complexes and cause the particles to become more stabilized (Fig. 2D). The zeta potentials of bare AuNPs and AuNPs with the antibody complex, antibody-capture DNA complex and antibody-capture DNA-barcode DNA complex were −23.1 mV, −31.3 mV, −38.7 mV, and −49.3 mV, respectively. The significance of the zeta potential lies in its value associated with the stability of colloidal dispersion and the successful formation of a complex. Among all of the samples, the AuNPs with the antibody-capture DNA-barcode DNA complex have a greater negative charge; thus, the samples containing the antibody-capture DNA-barcode DNA complex are more stable than the other samples.

### Optimization of the competitive bio-barcode amplification immunoassay

Changes in the concentration of immune reagents have a strong influence on detection. Therefore, choosing the optimal concentration of antibody and hapten is important in establishing a standard curve. Before optimizing the working concentration, we have determined the concentrations of antibody loading on AuNP and the concentrations of hapten loading on MNPs^26^. A measurement with UV/Vis spectroscopy was carried out to analyze the antibody and hapten content in the solution prepared before the immobilization reaction (original), after immobilization (supernatant) and the washing solutions(see Fig. S2 in the supplementary information). Based on the methods, the concentration of antibody and hapten bound onto the AuNPs and MNP as the stock solution was 30.2 mg L^−1^ and 64.2 mg L^−1^ respectively. Chessboard assay was used to optimize the working concentration of reagents. The concentration of hapten was 3.21, 1.60, 0.80, 0.64, and 0.43 mg L^−1^ and the concentration of antibody was 3.02, 1.51, 0.76, 0.38, 0.30 mg L^−1^. The results are shown in Table 1 and Fig. 3. Based on the high specificity and amplifying efficiency of real-time PCR, the optimal combination of reagents corresponded to the minimum Ct value^28^. Therefore, the optimal concentration of hapten was 0.80 mg L^−1^, and the optimal concentration of antibody was 1.51 mg L^−1^.

### Stability of the AuNPs probes

The gold nanoparticles (AuNPs) probe was designed by the assembly of antibody and alkylthiol-capped DNA (capture DNA) and bio-code DNA on gold nanoparticles. DNA and antibodies are vulnerable to lose its bioactivities because of the change of molecular conformation and surrounding environment in the synthetic process^29^,^30^,^31^. Therefore, it is necessary to assess the stability of the AuNPs probes.

The stability of the AuNPs probes was examined by using the same procedures described in Section of Competitive Bio-Barcode Amplification Immunoassay, except that the triazophos standard solution (5 ng/mL) was used instead of the sample solution. Figure 4 shows the stability of the dual-labeled AuNPs probe. The activity of the probe did not change significantly over 15 days, which shows that the probe can be used for repeated measurements during 15 days of storage at 4 °C.

### Cross-Reactivity

We investigated the specificity of the AuNPs probe based-immunoassay in the detection of target pesticides based on the optimized experimental conditions (Fig. 5). Ethyl parathion and chlorpyrifos were used as nonspecific antigens to demonstrate the specificity of the assay. We found that even at a high concentration of ethyl parathion (20 ng mL^−1^) and chlorpyrifos (20 ng mL^−1^), only minimal nonspecific signals were measured, whereas the Ct value for triazophos (0.5 ng mL^−1^) was higher than that of ethyl parathion and chlorpyrifos (Fig. 5). This finding shows that the AuNP probe-based immunoassay has excellent specificity in target detection.

### Standard curve

A standard curve of the bio-barcode amplification immunoassay (Fig. 6) was produced based on the optimized conditions given in Table 1 and Fig. 3. The linear equation is y = 3.78 x + 19.74 (R^2^ = 0.97). The linear range was from 0.04 ng mL^−1^ to 10 ng mL^−1^, and the limit of detection (LOD) was calculated using three standard deviations of blank samples (n = 20) plus the mean value, which resulted in 0.02 ng mL^−1^, which was 10–20 fold lower than ELISA^24^,^32^. Each concentration was measured 5 times in parallel.

### Recovery rates and accuracy

According to the MRLs of China and CAC (GB 2763–2014), blank samples (apple, orange, cabbage and rice) were spiked with the triazophos standard. The spiking concentrations were 10 μg kg^−1^, 50 μg kg^−1^, and 100 μg kg^−1^ respectively. The results are shown in Table 2. The recovery rates with the bio-barcode immunoassay were determined to range from 72.5% to 110.5% for triazophos, and the RSD was less than 20%. The results shows that the method meets the requirements for pesticide residue analysis. To validate the performance of the bio-barcode immunoassay, the concentrations of triazophos were simultaneously measured by GC-MS. The results indicated that the bio-barcode amplification immunoassay is a reliable immunoassay for the detection of triazophos.

### Analysis of real samples

The randomly selected 18 real samples of the apple, orange, cabbage, zucchini, and rice were purchased from Carrefour, Wal-Mart, other supermarkets, and farmers markets, totaling 72 samples. The real samples residue was listed in Table 3. Triazophos residue in apple, orange, cabbage and rice samples were lower than its MRLs of China (rice MRL: 0.1 mg/kg; cabbage MRL: 0.01 mg/kg).

## Conclusion

In conclusion, we have successfully established a competitive bio-barcode amplification immunoassay using a single monoclonal antibody followed with nanoparticles and real-time PCR in quantitative detection of small molecules. The detection limit of the proposed method was approximately 10–20 orders than that of the conventional ELISA method for triazophos. The competitive bio-barcode amplification immunoassay should have great potential applications in a diverse range of areas, such as medical diagnostics, environmental monitoring, and food safety.

## Materials and Methods

### Reagents and material

The triazophos standard, 2-(N-morpholino) ethanesulfonic acid (MES), 1-ethyl-3-(3-dimethylaminopropyl) carbodiimide hydrochloride (EDC), N-hydroxysuccinimide (NHS), N,N-dimethylformamide (DMF), N,N′-dicyclohexyl carbodiimide (DCC), Ovalbumin (OVA), Albumin from bovine serum (BSA), HAuCl_4_·3H_2_O and trisodium citrate were purchased from Sigma-Aldrich (St. Louis, MO, USA). Primary secondary amine (PSA) and C_18_ solid-phase extraction packing materials were purchased from Bonna-Agela Technologies (Tianjin, China). Carboxyl-functionalized magnetic nanoparticles (MNPs) and HPLC-grade acetonitrile and methanol were acquired from Thermo Fisher Scientific (MA, USA). All other chemicals and organic solvents, including Tris, hydrochloric acid, Tween 20, and peroxide, were of analytical grade or higher and were purchased from Beijing Chemical Industry Group Co., Ltd (Beijing, China). All oligonucleotides used in this work were ordered from Sangon Co., Ltd. (Shanghai, China).

### Preparation of gold nanoparticles (AuNPs) probes

**Figure 1:**
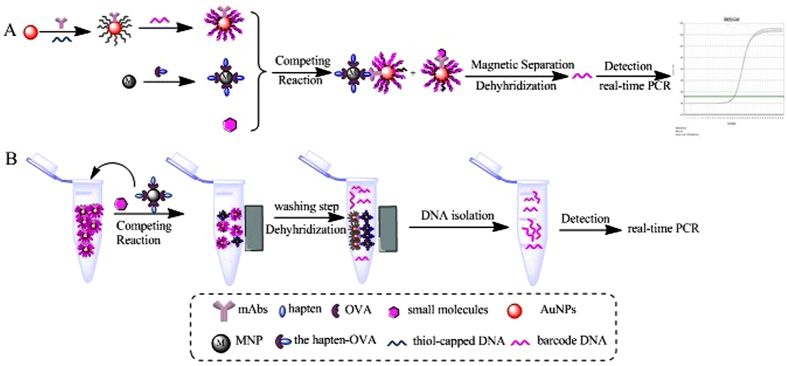
(**A**) Schematic illustration of the competitive bio-barcode amplification immunoassay based on nanoparticles. (**B**) The measurement process of the competitive bio-barcode amplification immunoassay.

**Figure 2:**
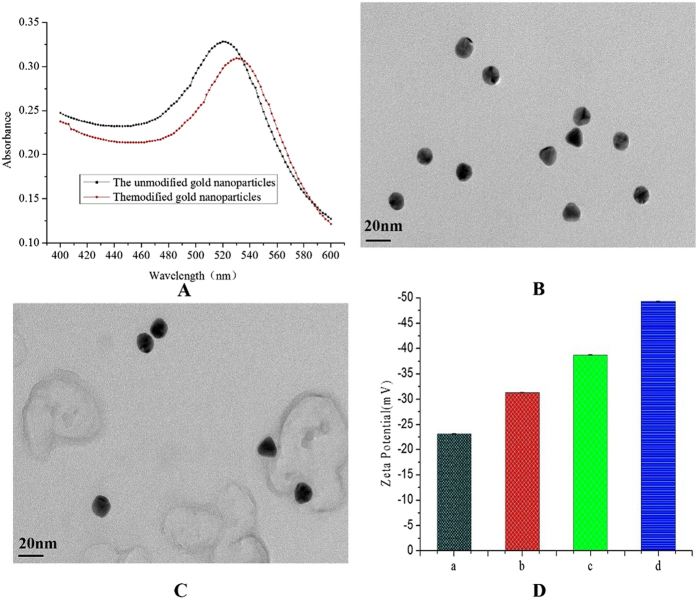
(**A**) UV/Vis spectra of the dispersion of the AuNPs probe and AuNPs solution. (**B**) TEM images of bare AuNPs. (**C**) AuNPs probes. (**D**) Zeta potentials (a) bare AuNPs, (b) AuNPs with the antibody complex, (c) AuNPs with the antibody-capture DNA complex, and (d) AuNPs with the antibody-capture DNA-barcode DNA complex.

**Figure 3:**
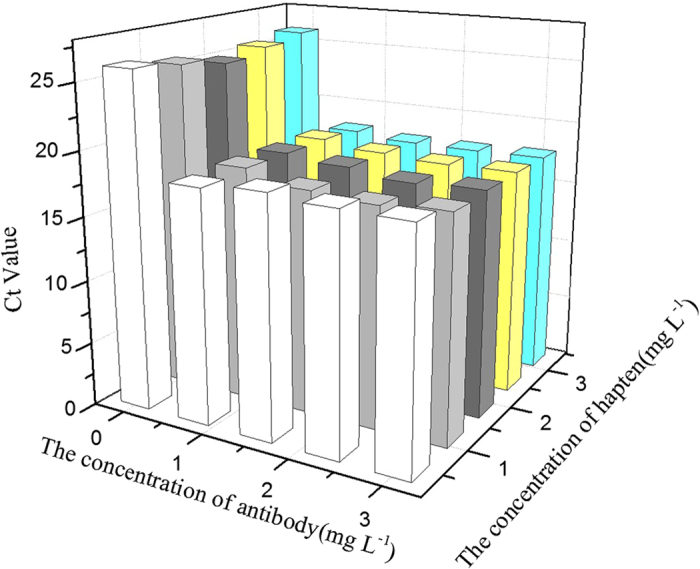
The 3D plot of optimization the working concentration.

**Figure 4:**
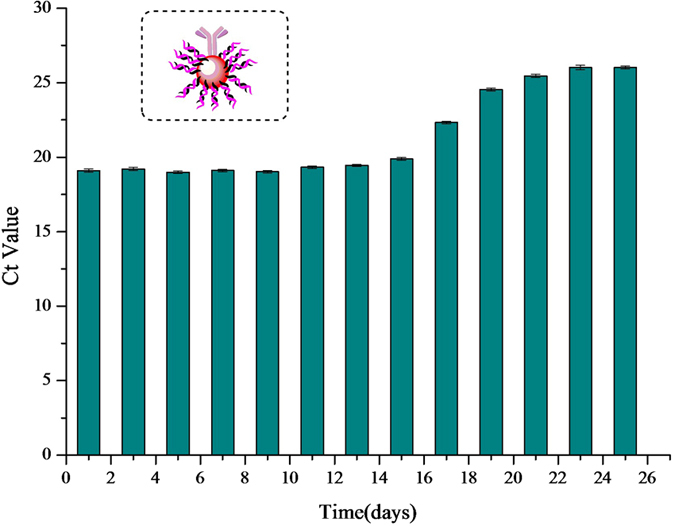
The stability of the AuNP probe.

**Figure 5:**
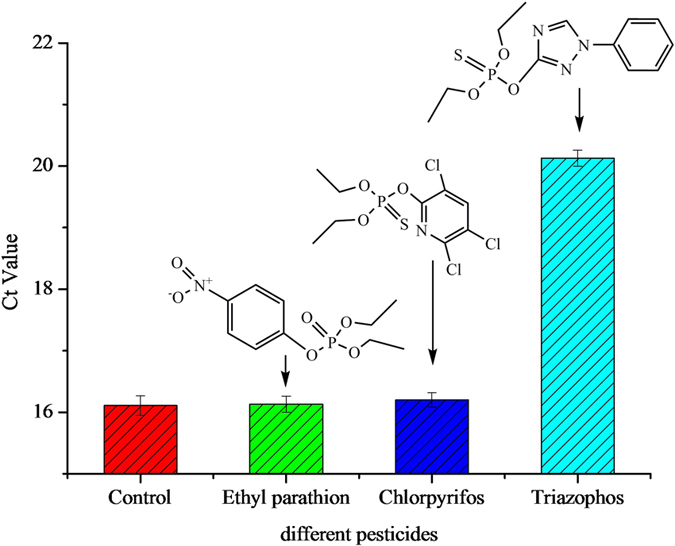
The specificity of the AuNP probe based-immunoassay in target pesticide detection.

**Figure 6:**
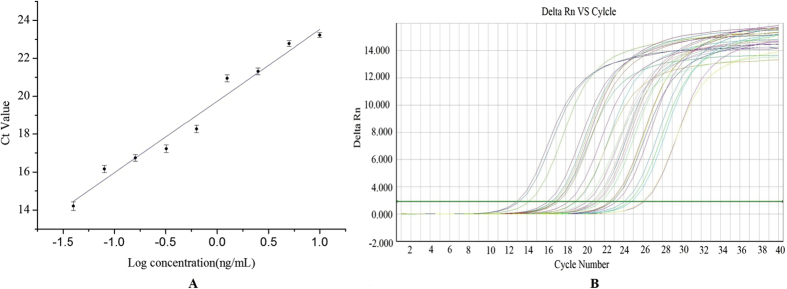
(**A**) The triazophos standard curves. (**B**) Amplification curves of dilution series of triazophos ranging from 0.04 ng mL^−1^ to 10 ng mL^−1^, and negative control (containing SYBR Green Real-time PCR Master Mix, but without template DNA).

Gold nanoparticles (AuNPs) probes were prepared following a previously reported procedure with a few modifications^28^,^33^. Figure 1A illustrates the schematic diagram of the fabrication of the AuNPs probe. A volume of 4 mL of AuNPs was adjusted to pH 8.5 with K_2_CO_3_. The AuNPs was incubated with 45 μg of monoclonal antibody (mAbs) for 30 min at room temperature under gentle shaking. A phosphate adjustment buffer was added to the AuNPs solution. The final phosphate concentration of 10 mM. Subsequently, 1 OD capture DNA was added and incubated for 16 h with gentle stirring at 4 °C. Then, NaCl (2 mol L^−1^) was added to the solution to obtain a final concentration of 0.15 M NaCl in a five-stepwise addition within 40 h. Next, 10% BSA were added to the mixture to obtain a final BSA concentration of 0.5% and incubated for 1 h. Then the solution was repeatedly centrifuged at 13000 rpm for 15 min at 4 °C. The particles were resuspended with 4 mL of PBS (0.01 mol L^−1^, pH 7.4) containing 0.15 mol L^−1^ NaCl after supernatant removed. Then, 1 OD barcode DNA, which is complementary to the capture DNA, was added and incubated for 4 h at room temperature. Another similar centrifugation procedure was performed to remove the excess barcode DNA and to obtain the modified AuNPs. Finally, the gold nanoparticles were resuspended in 1 mL of PBS containing 0.15 mol L^−1^ NaCl, 0.01% Tween-20. This solution was stored at 4 °C until use.

### Preparation of magnetic nanoparticles (MNPs) probes

Magnetic nanoparticles (MNPs) probes were functionalized with hapten-OVA conjugates by cross-linking carboxyl groups on the surface of the MNPs with amine groups in the OVA (Fig. 1A). First, 1 mL of MNPs was obtained and placed in a centrifuge tube, and then 1 mL of MES solution was added. The mixture was rotated for 10 seconds. Then, 1 mL of activation buffer was used to rewash the magnetic beads twice, and 500 μL of MES solution was added. Next, 500 μL of carbodiimide and 500 μL of N-hydroxysuccinimide were separately added and mixed for 30 min with slow rotation at room temperature. Subsequently, the MNPs were washed three times with PBST, and then 800 μg of hapten-OVA conjugates was added and incubated for 16–18 h at 37 °C with slight stirring. Afterwards, the MNPs probes were washed twice with PBST to remove excess hapten-OVA conjugates by a magnetic separation process. The nonspecific sites on the MNPs probes were blocked by incubating with PBS buffer (containing 2% BSA) at room temperature for 30 min with slight stirring. Finally, the MNPs probes were collected and stored at 4 °C for further use.

### Sample preparation

Blank samples (apple, orange, cabbage, rice) were stored in a refrigerator at −20 °C after mashed. For recovery studies^34^, apple, orange, cabbage and rice samples without target pesticide were spiked with triazophos. Solutions of the triazophos in methanol were added to 10 g of finely chopped apple, orange, cabbage, and rice. After the samples were set aside for 24 h, 3 mL of water was firstly added to a 50 mL plastic centrifuge tube for rice samples. The apple, orange, cabbage samples did not need to add water. Then acetonitrile extraction solution was added to obtain a constant volume of 10 mL for 10 min with vigorous shaking. Then, 4 g of anhydrous MgSO_4_ and 1 g of NaCl was added, vortexed immediately and centrifuged at 10,000 r/min for 5 min at 4 °C. Next, 2 mL of supernatant was accurately transferred to a microcentrifuge tube. Subsequently, 50 mg of PSA and 50 mg of C_18_ dispersive solid-phase extraction purifier were added, vortexed for 0.5 min and centrifuged at 10,000 r/min for 5 min at 4 °C. Then, 1 mL of the supernatant was concentrated by Termovap Sample Concentrator at 30 °C with nitrogen. Finally, the residue was dissolved in 10% methanol-PBS. The competitive bio-barcode amplification immunoassay was conducted to analyze the extract, and the recovery was determined using the standard curve obtained from the standards in methanol-PBS.

### Competitive Bio-Barcode Amplification Immunoassay

In a typical assay, the AuNPs probes were diluted with the binding buffer in Eppendorf tubes. Triazophos standards (10, 5, 2.5, 1.25, 0.63, 0.32, 0.16, 0.08, and 0.04 ng mL^−1^) in 10% (v/v) methanol-PBS or samples (20 μL) and 20 μL of MNPs probe solution were added to EP tubes incubated at 37 °C for 30 min. During this step, the antigen labeled on the surface of the magnetic nanoparticles and triazophos competed to combine with the antibody labeled on the surface of the gold nanoparticles. Then the complexes can be removed by magnetic separation and washed four times with 100 μL of PBS solution. Finally, 50 μL of H_2_O was added and stirred vigorously at 60 °C for 30 min to allow for full dehybridization. The complexes were removed again by magnetic separation, and the supernatant containing the barcode DNA was collected for quantification by PCR. Figure 1B represents the measurement process of the Competitive Bio-Barcode Amplification Immunoassay.

## Additional Information

**How to cite this article**: Du, P. *et al*. A Competitive Bio-Barcode Amplification Immunoassay for Small Molecules Based on Nanoparticles. *Sci. Rep.* **6**, 38114; doi: 10.1038/srep38114 (2016).

**Publisher's note:** Springer Nature remains neutral with regard to jurisdictional claims in published maps and institutional affiliations.

## Supplementary Material

Supplementary Information

## Figures and Tables

**Table 1:** Chessboard titration of the optimal concentration of hapten and antibody.

The concentration of hapten (mg L^−1^)	The concentration of antibody (mg L^−1^)					
	3.02	1.51	0.76	0.38	0.30	Negative control
3.21	17.75 ± 0.11	17.69 ± 0.13	18.06 ± 0.12	18.99 ± 0.13	19.52 ± 0.09	26.12 ± 0.07
1.60	17.65 ± 0.13	17.93 ± 0.08	18.31 ± 0.14	18.20 ± 0.12	19.48 ± 0.07	25.00 ± 0.10
0.80	17.74 ± 0.11	17.51 ± 0.09	18.49 ± 0.12	18.45 ± 0.13	19.71 ± 0.13	26.04 ± 0.08
0.64	17.99 ± 0.09	17.84 ± 0.10	18.29 ± 0.15	18.81 ± 0.10	19.97 ± 0.10	26.03 ± 0.06
0.43	18.26 ± 0.12	18.65 ± 0.14	18.22 ± 0.12	19.12 ± 0.13	19.31 ± 0.09	26.10 ± 0.09

**Table 2:** Reproducibility and recovery of triazophos from spiked samples (n = 5).

Sample	Spiked concentration (μg/kg)	Bio-bar code immunoassay (%)	RSD (% n = 5)	GC-MS (%)	Sample	Spiked concentration (μg/kg)	Bio-bar code immunoassay (%)	RSD (% n = 5)	GC-MS (%)
Apple	10.0	73.3	17.9	81.9	Cabbage	10.0	73.1	14.3	88.2
	50.0	84.1	10.8	87.6		50.0	99.2	14.8	91.4
	100.0	110.5	12.2	94.6		100.0	100.1	19.1	94.3
Orange	10.0	72.5	17.6	88.3	Rice	10.0	83.4	15.3	86.3
	50.0	89.0	18.7	91.4		50.0	89.2	13.1	93.5
	100.0	99.4	15.3	94.1		100.0	100.2	17.3	96.2

**Table 3:** The analysis of real samples residue.

Samples	Concentration (μg/kg)					
Apple	<LOD	<LOD	<LOD	<LOD	<LOD	<LOD
	0.05	<LOD	0.06	<LOD	<LOD	<LOD
	<LOD	<LOD	<LOD	0.04	<LOD	<LOD
Orange	0.12	<LOD	<LOD	<LOD	0.09	<LOD
	<LOD	0.13	<LOD	0.06	<LOD	<LOD
	<LOD	<LOD	<LOD	<LOD	<LOD	<LOD
Cabbage	0.14	<LOD	<LOD	0.15	0.09	<LOD
	<LOD	<LOD	0.12	<LOD	<LOD	<LOD
	<LOD	<LOD	<LOD	<LOD	<LOD	<LOD
Rice	<LOD	<LOD	0.13	<LOD	<LOD	<LOD
	0.08	<LOD	<LOD	<LOD	0.06	<LOD
	<LOD	<LOD	<LOD	<LOD	0.11	<LOD

The LOD of the method: 0.02 μg/kg.

## References

[b1] Cheng S. Y. . Sensitive Detection of Small Molecules by Competitive Immunomagnetic-Proximity Ligation Assay. Anal. Chem. 84, 2129–2132, doi: 10.1021/ac3001463 (2012). 22394090

[b2] Ding C. . An electrochemical immunoassay for protein based on bio bar code method. Biosens. Bioelectron. 24, 2434–2440, doi: 10.1016/j.bios.2008.12.023 (2009). 19167209

[b3] Seo S. H. . Highly sensitive detection of a bio-threat pathogen by gold nanoparticle-based oligonucleotide-linked immunosorbent assay. Biosens. Bioelectron. 64, 69–73, doi: 10.1016/j.bios.2014.08.038 (2015). 25194798

[b4] Tang D. . Low-cost and highly sensitive immunosensing platform for aflatoxins using one-step competitive displacement reaction mode and portable glucometer-based detection. Anal. Chem. 86, 11451–11458, doi: 10.1021/ac503616d (2014). 25329775

[b5] Chen J. . Electrochemical nanoparticle-enzyme sensors for screening bacterial contamination in drinking water. Analyst 140, 4991–4996, doi: 10.1039/c5an00637f (2015). 26042607 PMC4501873

[b6] Hauser C. A. . Amyloid-based nanosensors and nanodevices. Chem. Soc. Rev. 43, 5326–5345, doi: 10.1039/c4cs00082j (2014). 24781248

[b7] Lu Y. . Sandwich immunoassay for alpha-fetoprotein in human sera using gold nanoparticle and magnetic bead labels along with resonance Rayleigh scattering readout. Microchim. Acta 180, 635–642, doi: 10.1007/s00604-013-0965-z (2013).

[b8] Bao Y. P. . Detection of Protein Analytes via Nanoparticle-Based Bio Bar Code Technology. Anal. Chem. 78, 2055–2059 (2006). 16536446 10.1021/ac051798d

[b9] Stoeva S. I. . Multiplexed Detection of Protein Cancer Markers with Biobarcoded Nanoparticle Probes. J. Am. Chem. Soc. 128, 8378–8379 (2006). 16802785 10.1021/ja0613106

[b10] Duan R. X. . Electrochemiluminescence Biobarcode Method Based on Cysteamine-Gold Nanoparticle Conjugates. Anal. Chem. 82, 3099–3013 (2010). 20297795 10.1021/ac100018z

[b11] Nam J. M. . Nanoparticle-based bio-bar codes for the ultrasensitive detection of proteins. Science 301, 1884–1886, doi: 10.1126/science.1088755 (2003). 14512622

[b12] Nam J. M. . Detection of proteins using a colorimetric bio-barcode assay. Nat. Protoc. 2, 1438–1444, doi: 10.1038/nprot.2007.201 (2007). 17545980

[b13] Li W. . Multiplexed Detection of Cytokines Based on Dual Bar-Code Strategy and Single-Molecule Counting. Anal. Chem. 88, 1578–1584, doi: 10.1021/acs.analchem.5b03043 (2016). 26721199

[b14] Hee An, . Gold Nanoparticles-Based Barcode Analysis for Detection of Norepinephrine. J. Biomed. Nanotechnol. 12, 357–365, doi: 10.1166/jbn.2016.2185 (2016). 27305769

[b15] Georganopoulou D. G. . Nanoparticle-based detection in cerebral spinal fluid of a soluble pathogenic biomarker for Alzheimer’s disease. Proc. Natl. Acad. Sci. USA 102, 2273–2276, doi: 10.1073/pnas.0409336102 (2005). 15695586 PMC548981

[b16] Du L. . An ultrasensitive detection of 17beta-estradiol using a gold nanoparticle-based fluorescence immunoassay. Analyst 140, 2001–2007, doi: 10.1039/c4an01952k (2015). 25672478

[b17] Cao C. . Detection of avian influenza virus by fluorescent DNA barcode-based immunoassay with sensitivity comparable to PCR. Analyst 135, 337–342, doi: 10.1039/b916821b (2010). 20098768

[b18] Oh B. K. . A fluorophore-based bio-barcode amplification assay for proteins. Small 2, 103–108, doi: 10.1002/smll.200500260 (2006). 17193564

[b19] Zhou W. . Gold Nanoparticles for *In Vitro* Diagnostics. Chem. Rev. 115, 10575–10636, doi: 10.1021/acs.chemrev.5b00100 (2015). 26114396 PMC5226399

[b20] Zhan J. . Multi-class method for determination of veterinary drug residues and other contaminants in infant formula by ultra performance liquid chromatography-tandem mass spectrometry. Food Chem. 138, 827–834, doi: 10.1016/j.foodchem.2012.09.130 (2013). 23411184

[b21] Carter J. A. . A label-free, multiplex competitive assay for small molecule pollutants. Biosens. Bioelectron. 77, 1–6, doi: 10.1016/j.bios.2015.08.064 (2016). 26385730

[b22] Wang C. . An aptameric graphene nanosensor for label-free detection of small-molecule biomarkers. Biosens. Bioelectron. 71, 222–229, doi: 10.1016/j.bios.2015.04.025 (2015). 25912678 PMC4466219

[b23] Liang C. Z. . Enzyme-Linked Immunosorbent Assay Based on a Monoclonal Antibody for the Detection of the Insecticide Triazophos: Assay Optimization and Application to Environmental Samples. Environ. Sci. Technol. 41, 6783–6788 (2007). 17969695 10.1021/es070828m

[b24] Du P. . A rapid immunomagnetic-bead-based immunoassay for triazophos analysis. RSC Adv. 5, 81046–81051, doi: 10.1039/c5ra15106f (2015).

[b25] Zhai C. . Acetylcholinesterase biosensor based on chitosan/prussian blue/multiwall carbon nanotubes/hollow gold nanospheres nanocomposite film by one-step electrodeposition. Biosens. Bioelectron. 42, 124–130, doi: 10.1016/j.bios.2012.10.058 (2013). 23202341

[b26] Liu M. . Highly sensitive protein detection using enzyme-labeled gold nanoparticle probes. Analyst 135, 327–331, doi: 10.1039/b916629g (2010). 20098766

[b27] Huo Y. . A sensitive aptasensor for colorimetric detection of adenosine triphosphate based on the protective effect of ATP-aptamer complexes on unmodified gold nanoparticles. Biosens. Bioelectron. 78, 315–320, doi: 10.1016/j.bios.2015.11.043 (2016). 26638040

[b28] Yang G. . A gold nanoparticle based immunosorbent bio-barcode assay combined with real-time immuno-PCR for the detection of polychlorinated biphenyls. Sens. Actuators, B 214, 152–158, doi: 10.1016/j.snb.2015.02.128 (2015).

[b29] Vidal B. C. Jr . Stability and hybridization-driven aggregation of silver nanoparticle–oligonucleotide conjugates. New J. Chem. 29, 812, doi: 10.1039/b417683a (2005).

[b30] Herdt A. R. . DNA dissociation and degradation at gold nanoparticle surfaces. Colloids Surf., B 51, 130–139, doi: 10.1016/j.colsurfb.2006.06.006 (2006). 16879950

[b31] Qiao F. Y. . Antibody and DNA dual-labeled gold nanoparticles: Stability and reactivity. Appl. Surf. Sci. 254, 2941–2946, doi: 10.1016/j.apsusc.2007.10.046 (2008).

[b32] Jin R. Y. . Comparison of monoclonal antibody-based ELISA for triazophos between the indirect and direct formats. Food Agric. Immunol. 19, 49–60, doi: 10.1080/09540100801933454 (2008).

[b33] Kumar S. . Directional conjugation of antibodies to nanoparticles for synthesis of multiplexed optical contrast agents with both delivery and targeting moieties. Nat. Protoc. 3, 314–320, doi: 10.1038/nprot.2008.1 (2008). 18274533

[b34] Bruzzoniti M. C. . QuEChERS sample preparation for the determination of pesticides and other organic residues in environmental matrices: a critical review. Anal Bioanal Chem 406, 4089–4116, doi: 10.1007/s00216-014-7798-4 (2014). 24770804

