# Supplementary material for: A Competitive Bio-Barcode Amplification Immunoassay for Small Molecules Based on Nanoparticles
Source: Sci Rep. 2016 Dec 7;6:38114. doi: 10.1038/srep38114 (PMC5141433; doi:10.1038/srep38114)
Supplement: Supplementary Information [file srep38114-s1.doc]

**A** **Competitive Bio-Barcode** **Amplification Immunoassay for Small Molecules Based on** **Nanoparticles**

Pengfei Du, Maojun Jin^*^, Ge Chen, Chan Zhang, Zejun Jiang, Yanxin Zhang, Pan Zou, Yongxin She, Fen Jin, Hua Shao, Shanshan Wang, Lufei Zheng, Jing Wang^*^

Key Laboratory for Agro-Products Quality and Food Safety, Institute of Quality Standards & Testing Technology for Agro-Products, Chinese Academy of Agricultural Sciences, Beijing, 100081, China

*Address for Correspondence:

Dr. Maojun Jin

Tel: 010-82106570

E-mail: katonking@163.com

Prof. Dr. Jing Wang

Tel: 010-82106568

E-mail: [w_jing2001@126.com](mailto:w_jing2001@126.com)

**Buffers and solutions**

The following buffers were used in the experiments:

Phosphate-buffered saline (PBS) (pH 7.4, 0.01 mol L^-1^) was prepared as follows: 0.27 g of KH_2_PO_4_, 2.86 g of Na_2_HPO_4_•12H_2_O, 0.2 g of KCl, and 8.8 g of NaCl were weighed and dissolved into water and the pH value was adjusted to 7.4. Then, distilled water was added to a constant volume of 1000 mL, and the buffer was stored at 4ºC. The phosphate-buffered saline was used for the dilution of antibodies and in the preparation of standard solutions;

MES buffer (pH 6.0, 15 mmol L^-1^) was produced as follows: 0.32 g of MES (molecular mass 213.25) was dissolved into 90 mL of deionized water, and then the pH value was adjusted to 6.0. Then, distilled water was added to a constant volume of 100 mL, and the buffer was stored at 4ºC.

10 mmol L^-1^ EDC buffer was prepared by dissolving 10 mg of EDC (molecular mass 191.7) into 1 mL of deionized water prior to use.

10 mmol L^-1^ NHS buffer was made by dissolving a certain amount of NHS into deionized water before use.

PBST solution was obtained by adding Tween to phosphate buffer (pH 7.4, 0.01 mol L^-1^). Blocking solution was prepared by dissolving BSA in phosphate buffer (pH 7.4, 0.01 mol L^-1^). The PBST was used for washing.

The monoclonal antibodies (mAbs) and haptens of triazophos (THHe and THBu) were obtained from Zhejiang University. which were placed in 0.5 mL centrifuge tubes and stored at -18ºC for future use.

The sequences of the alkylthiol-capped DNA (capture DNA), the barcode DNA (signal DNA) ^1^, and the forward and reverse primers are provided in Table S1.

Table S1 Oligonucleotide and primer sequences used in this work.

| Capture DNA | 5′- TACGAGTTGAGACCGTTAAGACGAGGCAATCATGCAATCCTGAATGCG-A10-(CH2)6-SH-3′ |
| --- | --- |
| Signal DNA | 5′- CGCATTCAGGATTGCATGATTGCCTCGTCTTAACGGTCTCAACTCGTA-3′ |
| Forward primer | 5′-TTCAGGATTGCATGATTGCC-3′ |
| Reverse primer | 5′-ACGAGTTGAGACCGTTAAGACG-3′ |

**Synthesis of citrate-protected AuNPs**

A solution of 13-nm AuNPs was synthesized by chemical reduction according to a previously published protocol ^2,3^. Briefly, 250 mL of an aqueous solution of 1 mmol L^-1^ sodium citrate was rapidly injected into a boiling aqueous solution of HAuCl_4_ (100 mL, 1 mmol L^-1^) with vigorous stirring. The solution was maintained under boiling and stirring for an additional 20 min. An obvious color change of the reaction mixture was observed from transparent to dark blue to wine red. After being slowly cooled to room temperature, the particle solution was filtered through a 0.22-µm cellulose nitrate filter to remove any floating aggregates. The prepared AuNPs were characterized using transmission electron microscopy (TEM) and UV/Vis spectroscopy.

**Preparation of hapten-OVA (ovalbumin) conjugates**

The hapten-OVA conjugates were synthesized as previously described^4^. OVA solution (20 mg mL^-1^) was prepared in carbonate buffer solution for hapten conjugates. Hapten (0.25 mmol) was dissolved into 1 mL of DMF solution, and then, 60 μl of tributylamine and 30 μl of ethyl chloroformate were added and stirred for 1 h for the reaction. Next, 300 µL of the reaction solution was gently added to 6 mL of OVA solution, which was stirred at room temperature for 2 h. After the reaction, the solution was placed into a dialysis bag and dialyzed against distilled water three times. Over the next 3 days, PBS (0.01 mol L^-1^) was used to dialyze the solution with the refreshed dialysate 3-4 times per day. Finally, the same volume of glycerin was added, evenly mixed, and separately stored at 20ºC.

**Analysis**

**Real-time PCR**

Real-time PCR was performed with the 7300 Real Time PCR System (AB, California, USA) and SYBR Green PCR Master Mix (AB, California, USA) based on the instructions of the manufacturer. Barcode DNA was finally quantified by SYBR Green real-time PCR directly in PCR tubes. The amplification system was in a final volume of 25 μL containing 12.5 μL of SYBR Green Real-time PCR Master Mix, 2.0 μL of forward primer, 2.5 μL of reverse primer, and 8.0 μL of double-distilled water. PCR amplification was performed with an initial denaturation for 10 min at 95◦C, followed by 40 cycles of 15 s at 95◦C, 60 s at 60◦C, and 20 s at 75◦C with fluorescence detection at the last step of each cycle, and then holding at 95◦C for 3 min. After these steps, a melt procedure was conducted.

In the amplification curves of dilution series of triazophos, The Ct (threshold cycle number) is the fractional cycle number at which the fluorescence signal reaches an arbitrary but defined threshold value within the early exponential phase of the reaction. Ct values are proportional to the logarithm of the initial copy numbers of the target DNA. Rn was the fluorescence emission intensity of the reporter dye divided by the fluorescence emission intensity of the passive reference dye.Rn+ was the Rn value of a reaction containing all components, including the template. Rn- was the Rn value of an un-reacted sample. ΔRn (delta Rn) was the magnitude of the signal generated by the given set of PCR conditions. The ΔRn value is determined by the following formula: (Rn+) – (Rn-). Standard A sample of known concentration used to construct a standard curve. By running standards of varying concentrations, you create a standard curve from which you can extrapolate the quantity of an unknown sample.

**GC-MS**

Gas chromatography-tandem mass spectrometry (GC-MS) (Agilent 7890–5975C, USA)was performed using the following conditions: column: Agilent DB-5MS (30 m × 250 μm × 0.25 μm) silica capillary column; oven temperature procedure: 60ºC maintained for 4 min, heated at 30ºC/min to 180ºC, and then heated to 250ºC at 10ºC/min and maintained for 4 min; carrier gas: helium, purity ≥ 99.999%, constant voltage mode, and 7.136-psi pressure; injection temperature: 220ºC; injection volume: 1 μL; injection mode: splitless injection, with the by-pass valve and septum purge valve opened 1.0 min later; electron impact ionization (EI): 70 eV; ion source temperature: 200ºC; GC-MS interface temperature: 250ºC.

The limit of detection (LOD) of GC-MS were defined as the concentration at 3 times the signal intensity of noise in the spiked samples. So the scope of triazophos detection by GC-MS in our work was from 5.2 ng mL^-1^ to 800 ng mL^-1^ and the detection limit (LOD) is 1.86 ng mL^-1^.

**Optimization of the mAbs concentration for the AuNPs probes**

The optimal concentration of mAbs for the conjugation of gold nanoparticles (AuNPs) probes was determined. A preliminary titration was carried out to verify the optimal concentration of mAbs for the conjugation of AuNPs probes. The pH of the AuNPs solution was adjusted with either HCl or K_2_CO_3_ (buffers could not be used because they would cause aggregation of the AuNPs) to 8.5. Next, 100 μL from each solution was transferred to 8 wells of a 96-well plate. Then, different volumes (1 μl, 2 μl, 3 μl, 4 μl, 5 μl, 6 μl, 7 μl) of mAbs at a fixed concentration of 450 μg/mL were added to each well. After 15 min, 10 μL of 10% NaCl solution was added to each solution to stimulate gold aggregation. Finally, a spectrophotometric measurement was carried out to record the absorbance at 400 and 580 nm (Fig. 1). The spectra of the probe solutions became increasingly similar to that of pure AuNPs. On this basis, the actual amount (6 μl) plus 20% of the desired antibody was stable. Therefore, we chose 7.2 μl as the optimal volume of mAbs.


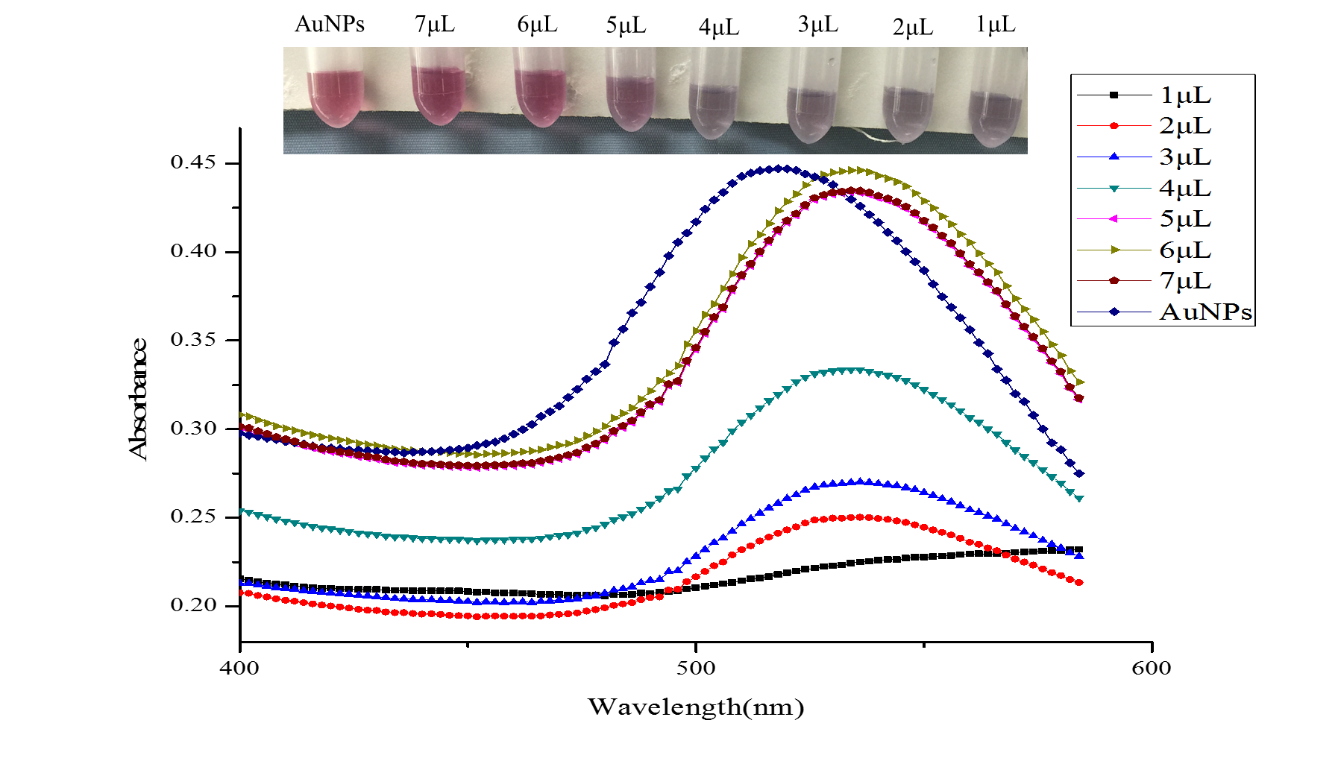


Fig. S1. Optimization of the concentration of triazophos mAbs for AuNPs probes


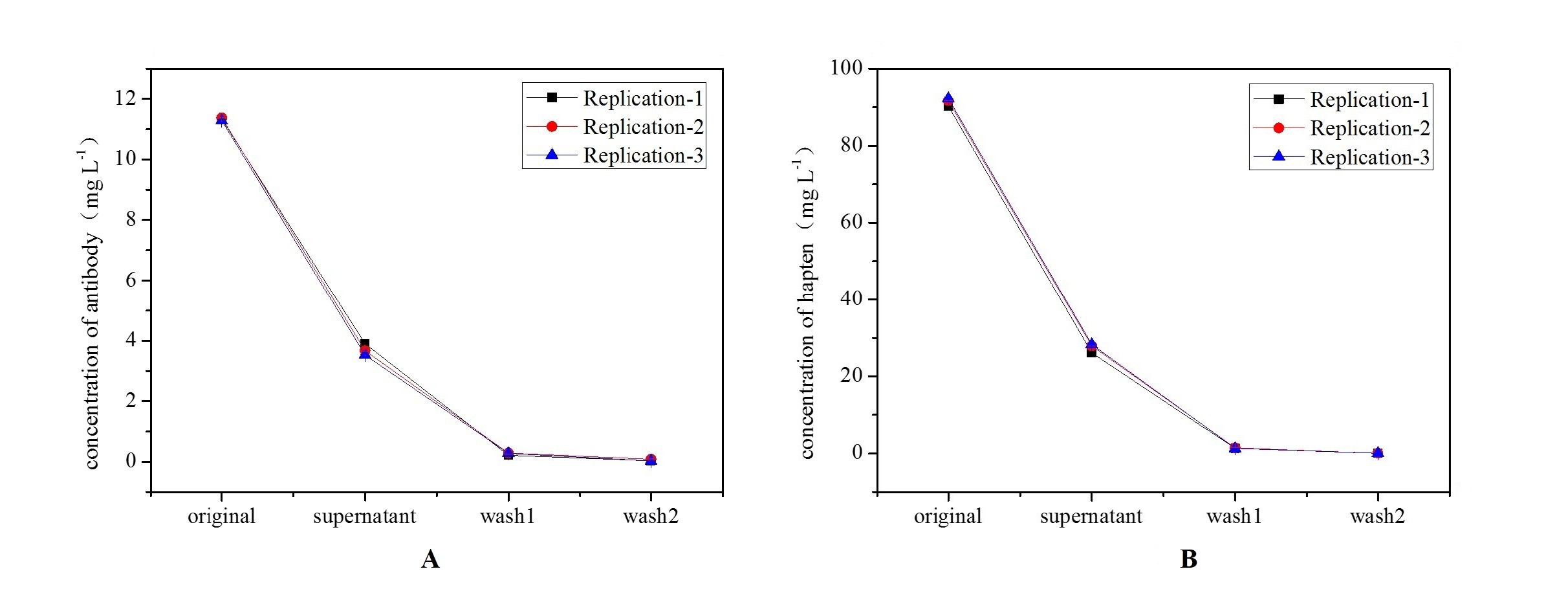


Fig. S2 The concentration of antibody loading on AuNPs (A) and the concentration of hapten loading on MNPs (B). The concentration of antibody and hapten in these solutions was determined via measuring UV/Vis absorbance.

**References**

1 Georganopoulou, D. G. *et al.* Nanoparticle-based detection in cerebral spinal fluid of a soluble pathogenic biomarker for Alzheimer's disease. *Proc. Natl. Acad. Sci. U. S. A.* **102**, 2273-2276, doi:10.1073/pnas.0409336102 (2005).

2 Nam, J. M. *et al.* Nanoparticle-based bio-bar codes for the ultrasensitive detection of proteins. *Science* **301**, 1884-1886, doi:10.1126/science.1088755 (2003).

3 Nam, J. M. *et al.* Detection of proteins using a colorimetric bio-barcode assay. *Nat. Protoc.* **2**, 1438-1444, doi:10.1038/nprot.2007.201 (2007).

4 Du, P. *et al.* A rapid immunomagnetic-bead-based immunoassay for triazophos analysis. *RSC Adv.* **5**, 81046-81051, doi:10.1039/c5ra15106f (2015).
